# Language Intervention in Down Syndrome: A Systematic Literature Review

**DOI:** 10.3390/ijerph19106043

**Published:** 2022-05-16

**Authors:** Esther Moraleda-Sepúlveda, Patricia López-Resa, Noelia Pulido-García, Soraya Delgado-Matute, Natalia Simón-Medina

**Affiliations:** 1 Facultad de Ciencias de la Salud, Universidad de Castilla-La Mancha, 45600 Talavera de la Reina, Spain; patricia.lopezresa@uclm.es (P.L.-R.); noeliapulidogarcia@hotmail.com (N.P.-G.); sorayateresa.delgado@alu.uclm.es (S.D.-M.); 2 Facultad de Educación de Toledo, Universidad de Castilla-La Mancha, 45005 Toledo, Spain; natalia.simon@uclm.es

**Keywords:** intervention, language, Down syndrome, systematic review

## Abstract

Language is one of the most affected areas in people with Down syndrome and is one of the most influential throughout their development. That is why the linguistic difficulties presented by this group are susceptible to treatment through different specific interventions. However, little emphasis has been placed on the effectiveness and importance of this type of intervention in improving their language skills. Therefore, this work aimed to carry out a systemic literature review of language intervention programs that have been carried out in the last 20 years. To this end, a total of 18 articles were analyzed in which the effectiveness of different types of treatment related to oral language, written language and communication, in general, was studied, using the guidelines of the PRISMA Statement and the COSMIN methodology. The results highlight that language intervention improves linguistic levels in people with Down Syndrome. Most of the research focuses on early interventions and interventions carried out through individual sessions. Nevertheless, the data are unanimous in considering the efficacy and effectiveness of the proposed treatments for improving the language skills of people with Down syndrome. Thus, linguistic intervention is a fundamental area of work throughout the lives of people with Down syndrome.

## 1. Introduction

Down syndrome (DS) consists mainly of an alteration in the number of chromosomes, which can lead to a genetic disorder. It is the most frequent aneuploidy in live newborns and, as we said, the main and most frequent genetic cause of intellectual disability [1,2,3]. This genetic modification causes alterations in the development and function of organs and systems, both in the prenatal and postnatal stages [4,5]. Despite the great variability in DS cases, the characteristic phenotype of DS causes alterations in the development and acquisition of language [6,7,8].

From all the above, it is easy to infer that people with DS are going to develop problems in both language and communication that are generally due to problems in development [9]. For this reason, it is important to comprehend and study the language development of children with DS as well as each of the components comprising it, thus understanding and “discovering” the aspects that need to be influenced by the intervention [10]. Regarding the development of language, the results of research conducted so far have been interpreted in a variety of manners. Some authors support the hypothesis of a slower pace in the language acquisition process compared to children with Typical Development (TD) [11,12]. Other authors emphasize the differences in language acquisition between children with DS and children with TD to conclude that language development follows a different course of development from the very beginning [13]. Along the same lines, several studies have shown that the language of children with DS comprises a qualitatively different pattern compared to children with TD, mainly associated with their phenotype [6,14,15,16].

In any case, language skills in people with DS are not uniform and show great individual variability [17]. These children show different specific dissociations in the various domains and subdomains of language. For example, they develop better comprehension than production [18,19,20,21] and as regards production, they present a better lexical level than morphosyntactic skills [22,23,24,25,26].

Given the characteristics described above, people with DS are invariably candidates for speech therapy interventions. Language intervention for people with DS should try to improve functioning in communication, academic, social and professional areas [27]. Although most of the interventions put forward in this regard are developed in the early stages of language development [28,29,30], the language of people with DS can improve [31] and continue developing beyond childhood [32], especially if they continue to work with an appropriate language intervention program [33]. Therefore, speech therapy intervention is effective throughout childhood [34] and should be considered as part of a comprehensive intervention for people with DS [35,36].

Given the importance of language work in people with Down syndrome, the objective of this research was to review the scientific literature on the effectiveness of language intervention in this population.

## 2. Materials and Methods

### 2.1. Strategy for the Identification of Articles

To obtain a broader knowledge of the effectiveness of speech therapy interventions in people with DS, a systematic review of the literature was carried out following the guidelines of the PRISMA Statement [37] and the COSMIN methodology [38]. The literature search related to the research goal was carried out using five databases: MEDLINE, Web of Science, PsycINFO, Scopus and PubMed. Likewise, to reduce publication bias, the “Education Resources Information Center” (ERIC) database was included.

Moreover, the search strategy employed was put forward to efficiently determine the relevant documents in terms of the research that this review concerns. For this purpose, the following DeCS descriptors were used: “Down’s Syndrome”, “Rehabilitation of Speech and Language Disorders”, “Speech and Language Intervention”, “Speech and Language Therapy” and the Boolean operators “AND” and “OR” in order to be able to obtain all possible combinations.

### 2.2. Strategy for the Selection of Articles

The following criteria were used for the selection of articles: (1) published between 2000 and 2020, (2) written in Spanish or English and (3) comprising empirical studies that address speech therapy intervention in DS.

In this manner, the search process was carried out entirely in English and Spanish and the search fields were limited to the title, keywords and abstract. Articles that were not relevant to the review at hand, articles that dealt solely and exclusively with pharmacological treatment and articles not published in the chosen languages were excluded. Furthermore, those publications included in the period from 2000 to 2020 were included when their typology related to empirical research addressing or conducting speech therapy interventions in DS. The search was not limited by design type. To complement the search, a review of the bibliography contained in the selected articles was carried out.

Regarding the selection process, a taxonomic selection organized into three stages was conducted. The first stage was based on reading the titles of the results obtained in the various databases to choose those related to the goal of the review. The second involved reading the summaries to analyze whether they met the inclusion and exclusion criteria. The last stage focused on qualitative analysis through a comprehensive reading of the pre-selected papers [39] and the COSMIN checklist manual for conducting systematic reviews [37]. Subsequently, data collection was carried out, extracting pertinent information from each selected article to conduct this review.

### 2.3. Strategy for the Analysis of Scientific Evidence

The strategy followed for the analysis of scientific evidence aimed to facilitate the coding process of the characteristics and results of the different studies. More specifically, the following variables were coded: citation in APA format, type of study, study goals, sample size and average age, area of language on which intervention is conducted, type of speech therapy intervention (individual or group), frequency of the intervention sessions, results and main conclusions drawn.

A total of 2770 documents were identified by applying the search strategy explained above. No additional articles were added after a review of article references. Of the 2770 articles, 1899 were eliminated due to being duplicated, triplicated or quadrupled.

**Figure 1:**
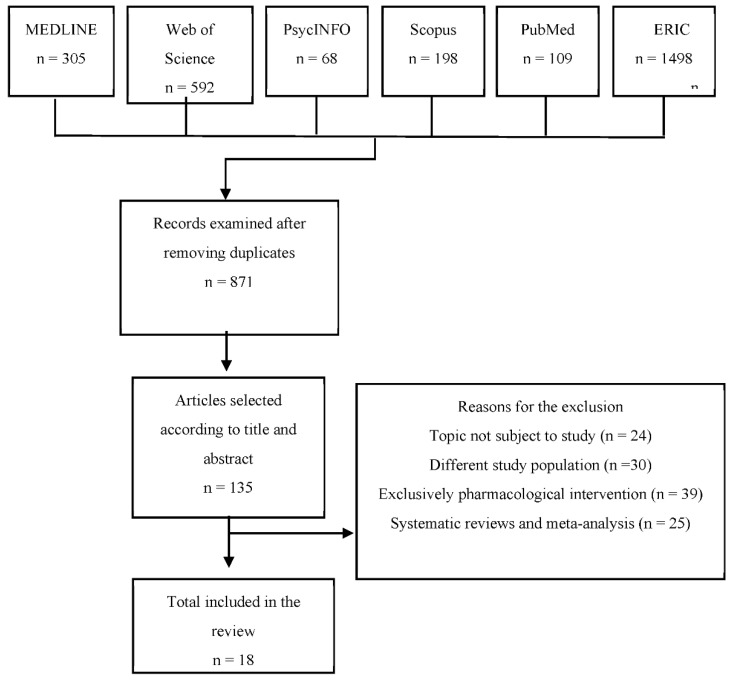
Flowchart of the information obtained through the different phases of the systematic review.

After screening by reading the title and abstract and applying the inclusion and exclusion criteria, a total of 136 articles were obtained, of which 24 were excluded because they did not focus on the object of study, 30 because they did not include people with DS in their sample, 39 because they involved an entirely pharmacological intervention and 25 for being systematic reviews or meta-analyses. Therefore, the final selection consisted of 18 articles. This entire process is detailed in Figure 1.

## 3. Results

Quantitative and qualitative analyses of the data were performed, and the average size of the total sample was computed. In addition, the average age was determined by calculating a weighted average based on the sample size. Lastly, the methodological quality of the included studies (Table 1) was evaluated using the COSMIN checklist [38] for systematic reviews.

The COSMISN risk of Bias contains ten boxes with standards for PROM development (box 1) and for nine measurement properties: Content validity (box 2), Structural validity (box 3), Internal consistency (box 4), Cross cultural validity/measurement invariance (box 5), Reliability (box 6), Measurement error (box 7), Criterion validity (box 8), Hypotheses testing for construct validity (box 9) and Responsiveness (box 10).

Finally, the study results were analyzed to individually assess bias and make a selection for subsequent data synthesis.

As reflected in Table 2, the final sample of this review consisted of 427 participants aged between 2 and 24 years (X = 6.96, SD = 6.06). Of the 427 participants, 416 had a diagnosis of DS (X = 6.82, SD = 5.66), and 11 were neurotypical. Only the studies by Burgoyne, Duff, Clarke, Buckley, Snowling and Hulme [51], Carlstedt, Henningsson and Dahllöf [48], Goetz et al. [50], Naess [46] and Sepúlveda, López-Villaseñor and Heinze [31] included a comparison control group and of these, only the studies by Carlstedt, Henningsson and Dahllöf [48] and Naess [46] included a neurotypical population group.

Regarding gender, the different studies presented a similar proportion of men and women. In this sense, 46.05% of the participants were women, and 53.95% were men. The studies of Barbosa, Lima, Alves and Delgado [45], Camarata, Yoder and Camarata [41], Finestack, O’Brien, Hyppa-Martin and Lyrek [49], Regis, Lima, Almeida, Alves and Delgado [33], Yoder, Woynaroski, Fey and Warren [52] and Yoder, Woynaroski, Fey, Warren and Gardner [29] did not specify the proportion of men and women in the sample.

As Table 3 illustrates, the research included in this study had very diverse specific objectives depending on the different areas of language. Most of the studies found in this regard were oriented—broadly speaking—to speech therapy intervention in oral language, written language and communication in DS.

The foregoing—considering the fact that the research included in this review was conducted with English (primarily) and Spanish-speaking populations—gives rise to a variety of assessment instruments chosen according to the goal and the mother tongue of the target study population (Table 4).

Regarding the specific results of the area related to metaphonological skills and literacy, the research by Burgoyne et al. [43,51] and Lemos et al. [42,47] showed that the explicit work of phoneme identification, syllable segmentation and phoneme substitution and addition significantly improved the decoding process that occurred in word reading. In this vein, Goetz et al. [50] also showed that the effectiveness of the intervention on metaphonological and decoding skills remained stable up to 5 months after the intervention ended. Furthermore, the study by Naess [46] showed that children with DS obtained a greater improvement in phonological, syllabic and rhyming awareness than their age peers with normal development as a result of formal education. In addition, Burgoyne et al. [43] and Van Bysterveldt, Gillon and Foster-Cohen [40] showed that explicit and structured work on phonological skills has a positive impact—in addition to reading—on the active lexicon and articulation, respectively.

Concerning oral language, the various papers reviewed offer an overview of the efficacy of speech therapy intervention in the comprehension and expression aspects of language. At a comprehension level, the study by Camarata, Yoder and Camarata [41] concludes that oral comprehension improves after the application of a naturalist intervention program. On the other hand, Linn et al. [44] reported improved sentence comprehension and passive vocabulary as a result of a family-focused intervention program. Continuing with the expressive level, the works reviewed show an increase in the Mean Length of Utterance [41,49], an increase in the active lexicon [29,46,51], a greater use of gestures to support communication and a substantial improvement in morphology, syntax and semantics [31]. In addition, the work of Wright, Kaiser, Reikowsky and Roberts [28] evidenced the efficacy of using Signed Augmentative Communication Systems in articulation-based speech therapy intervention and social communication. Lastly, Regis, Lima, Almeida, Alves and Delgado [33] concluded that speech therapy intervention favored the acquisition of skills closely related to language development and acquisition, such as imitation, communicative intention or designation.

Specifically concerning communicative and pragmatic skills, the work of Barbosa, Lima, Alves and Delgado [45] concluded that there was an improvement in social communication, autonomy and independence in the workplace of subjects with DS after the implementation of a naturalistic speech therapy intervention program. In this vein, Carlstedt, Henningsson and Dahllöf [48] showed that speech therapy intervention implemented simultaneously with the use of palatal expanders not only favored speech intelligibility but also increased the communicative intent of people with DS.

## 4. Discussion

The review of speech therapy intervention programs carried out with people with DS has yielded certain relevant findings. The first is that, in most cases, there are significant improvements after language treatment, so the need to carry out systematized interventions at the language level should continue to be insisted on throughout the entire development stage, as has been suggested by authors such as Rondal and Buckley [54]. However, it should be noted that there is still little research based on the effectiveness of intervention programs and strategies that improve the linguistic traits of people with DS [7,32] and this prevents us from generalizing their effectiveness. In addition, most were made with very small samples and focused primarily on children. There is hardly any literature on the efficacy of linguistic intervention in adults with DS.

In summary, all the studies concluded that the participants with DS obtained significant improvements in the areas assessed after receiving the systematized speech therapy intervention, regardless of the duration and frequency of the applied treatments. However, the study by Martín-Urda, Carchenilla and Moraleda [53] suggested that the effectiveness of an intervention program can be affected by the degree to which it has been systematized. In this vein, studies such as Burgoyne et al. [51] and Goetz et al. [50] showed that, although it is true that people with DS obtain a significant improvement after participating in an intervention program, the range of improvement grows when the intervention is conducted more frequently—in terms of sessions per week. Likewise, Carlstedt, Henningsson and Dahllöf [48] and Sepúlveda, López-Villaseñor and Heinze [31] concluded that, in addition, the suggested interventions were effective compared to other control groups, obtaining improvements with respect to the neurotypical population in the case of the former and regarding the DS population who were not administered the intervention program in question in the case of the latter.

The second finding is that, after the review, it was observed that most of the work was done with English-speaking children and, for this reason, the results and effectiveness thereof may be different depending on the language. According to Vicari et al. [13], it would be necessary to conduct research using other complex languages, such as Spanish. Moreover, the reviewed intervention may be very different in the various consulted research studies, depending on the areas on which the intervention focuses, since there are programs that start from a global stimulation of language [33], and others that focus expressly on specific areas, such as phonological awareness [46,47] or pragmatics [44,45]. It seems, therefore, that there is no consensus or continuity when proposing exhaustive interventions concerning different linguistic areas.

On the other hand, it is important to note that most of the research consulted considers the intervention from an individual perspective. This aspect is decisive when it comes to underlining and understanding that individualization in people with some kind of disability (in this case, DS) is essential when working toward the goals set [18,40,55,56,57]. Except in very specific cases, it seems clear that working with the specific needs of each person increases the effectiveness of the intervention.

In this prioritization of the intervention’s specific goals, the priorities of the family, the severity of the deficit and the importance of learning for functionality in educational and social contexts should also be taken into account [58,59,60]. Similarly, knowledge of the cognitive–behavioral phenotype of DS, such as the neurocognitive profile and developmental trajectory, can also guide intervention practices [61].

## 5. Conclusions

In conclusion, this work aimed to carry out a systemic literature review of the 13 language intervention programs that have been carried out in the last 20 years. There is a relatively high level of knowledge about the development of language in people with DS and its difficulties, which is of great help and lays the foundation for directing the work of therapists and teachers [54]. A fundamental concern derived from this has been the adoption of a perspective that covers the entire life cycle of the person with DS, bearing in mind that language intervention is tailored to age and takes into account the communication needs of individuals with regard to their surroundings. Therefore, it is considered necessary to continue incorporating language-related professional practices—carried out from an educational or clinical approach in people with DS—into research, in order to continue improving this group’s skills, and, consequently, their quality of life.

The focus of this work consists of emphasizing the importance of speech therapy intervention and highlighting the lack of studies about the evaluation of the developed linguistic treatments. Therefore, we consider it essential for professionals and researchers to continue checking the linguistic intervention methods that are being finished.

Regarding the limitations of this review, it should be considered that the objective of the different studies reviewed is focused on different linguistic areas. In addition, we can find results in different languages, such as English, Spanish or Portuguese, with a different complexity, which could explain their variability.

## Figures and Tables

**Table 1:** Results of the evaluation of the quality of the studies.

Research	1	2	3	4	5	6	7	8	9	10	Evaluation
van Bysterveldt, Gillon and Foster-Cohen (2010) [40]	+	+	+	+	+	−	+	+	+	?	High
Camarata, Yoder and Camarata (2006) [41]	+	+	+	+	?	−	+	+	+	−	Medium
Lemons, King, Davidson, Puranik, Al Otaiba and Fidler (2018) [42]	+	?	+	+	+	−	+	+	+	−	Medium
Burgoyne et al. (2012) [43]	?	+	+	+	+	−	+	+	+	?	Medium
Linn et al. (2019) [44]	+	+	+	+	?	−	+	+	+	−	Medium
Barbosa, Lima, Alves and Delgado (2018) [45]	+	+	+	+	+	−	?	+	+	−	Medium
Naess (2016) [46]	+	+	+	+	+	−	?	+	+	−	Medium
Lemons et al. (2015) [47]	?	+	+	+	?	−	+	+	+	?	Medium
Sepúlveda, López-Villaseñor and Heinze (2013) [31]	+	?	+	+	?	−	+	+	+	−	Medium
Wright, Kaiser, Reikowsky and Roberts (2013) [28]	+	+	+	+	+	+	?	+	+	+	High
Yoder, Woynaroski, Fey, Warren and Gardner (2015) [29]	+	+	+	+	−	+	−	+	+	?	Medium
Carlstedt, Henningsson, Dahllöf (2003) [48]	?	+	+	+	+	−	−	+	−	+	Low
Finestack, O’Brien, Hyppa-Martin and Lyrek (2017) [49]	+	+	+	+	+	+	?	−	+	?	Medium
Goetz, Hulme, Brigstocke, Carroll, Nasir and Snowling (2008) [50]	+	+	+	−	+	+	+	−	+	?	Medium
Burgoyne, Duff, Snowling, Buckley and Hulme (2013) [51]	?	+	+	−	+	+	+	+	−	+	Medium
Regis, Lima, Almeida, Alves and Delgado (2018) [33]	+	+	+	+	?	+	?	+	−	+	Medium
Yoder, Woynaroski, Fey and Warren (2014) [52]	−	+	−	−	+	+	+	+	+	+	Medium
Martín-Urda, Carchenilla and Moraleda (2019) [53]	+	−	?	+	+	+	?	+	+	+	Medium

+ positive; − negative; ? Not proceed.

**Table 2:** Sociodemographic variables of the studies reviewed.

Authors	Sample Description			Control Group	Study Component
	N	Average Age	Sex (F/M)		
van Bysterveldt, Gillon and Foster-Cohen (2010) [40]	10	4.91	5/5	No	Speech and phonological awareness
Camarata, Yoder and Camarata (2006) [41]	6	5.7	Not specified	No	Grammar and speech
Lemons, King, Davidson, Puranik, Al Otaiba and Fidler (2018) [42]	6	8.1	5/1	No	Phonological awareness
Burgoyne et al. (2012) [43]	57	6.59	28/29	Yes	Language and literacy
Linn et al. (2019) [44]	21	2.25	11/10	No	Language development
Barbosa, Lima, Alves and Delgado (2018) [45]	5	24	Not specified	No	Pragmatics
Naess (2016) [46]	43	6.3	22/21	No	Phonological awareness
Lemons et al. (2015) [47]	5	7.34	23	No	Phonological awareness
Sepúlveda, López-Villaseñor and Heinze (2013) [31]	20	10.58	9/11	Yes	Morphosyntax
Wright, Kaiser, Reikowsky and Roberts (2013) [28]	4	2.08	2/2	No	Expressive language
Yoder, Woynaroski, Fey, Warren and Gardner (2015) [29]	64	1.83	Not specified	No	Expressive lexicon
Carlstedt, Henningsson, Dahllöf (2003) [48]	20	2	8/12	Yes	Speech and pragmatics
Finestack, O’Brien, Hyppa-Martin and Lyrek (2017) [49]	4	11.9	Not specified	No	Narrative skills
Goetz, Hulme, Brigstocke, Carroll, Nasir and Snowling (2008) [50]	15	10.3	8/7	No	Literacy
Burgoyne, Duff, Snowling, Buckley and Hulme (2013) [51]	10	8.33	2/8	No	Phonological awareness
Regis, Lima, Almeida, Alves and Delgado (2018) [33]	49	16.25	Not specified	No	Language development
Yoder, Woynaroski, Fey and Warren (2014) [52]	76	2.08	Not specified	Yes	Active lexicon
Martín-Urda, Carchenilla and Moraleda (2019) [53]	12	16.78	5/7	No	Oral language

**Table 3:** Study goals and intervention design of the studies consulted.

Authors/Year	Study Goal	Intervention Design
van Bysterveldt, Gillon and Foster-Cohen (2010) [40]	To analyze the efficacy of an intervention approach in the development of speech and phonological awareness in subjects with DS at the preschool age.	1 session per week of 20 min of speech therapy where computer-based learning was used.
Camarata, Yoder and Camarata (2006) [41]	To highlight the benefits of grammatical and speech intervention in the social inclusion of people with DS.	Naturalistic intervention based on restructuring. Daily intervention.
Lemons, King, Davidson, Puranik, Al Otaiba and Fidler (2018) [42]	To assess the potential efficacy and feasibility of early intervention for children with DS.	4 interventions a week of between 20 and 40 min
Burgoyne et al. (2012) [43]	To assess the effects of language and literacy intervention in children with DS.	5 sessions of 40 min of intervention per week (20 weeks)
Linn et al. (2019) [44]	To describe the type of communicative behaviors before and after undergoing a training program in gestural communication based on “signs, words and games” workshops from the “baby signs” program.	Intervention 7 weeks. 1 workshop per week.
Barbosa, Lima, Alves and Delgado (2018) [45]	To analyze the contributions of speech therapy interventions in the integration of young people with DS in the workplace, with reference to their professionalization.	Naturalistic intervention at work. Daily intervention, 5 days a week.
Naess (2016) [46]	To analyze phonological awareness skills in children with DS compared to TD	Daily school intervention, 5 days a week.
Lemons et al. (2015) [47]	To determine if the adaptation of a phonological awareness program would improve the learning process of children with DS, the sounds of letters and words.	Manipulative intervention five days a week.
Sepúlveda, López-Villaseñor and Heinze (2013) [31]	To determine if subjects with DS can improve in the morphosyntactic area	30 sessions of 30 min of intervention distributed over three and a half months. Participants received two sessions per week.
Wright, Kaiser, Reikowsky and Roberts (2013) [28]	To analyze the effects of a bimodal Augmentative and Alternative Systems of Communication on the expressive language of young children with DS	2 intervention sessions of 30 min per week. Game-based learning
Yoder, Woynaroski, Fey, Warren and Gardner (2015) [29]	To determine the effectiveness of the frequency of speech therapy intervention in the lexical component of children with DS	Attending the Early Attention Service (Servicio de Atención Temprana). 1 weekly session
Carlstedt, Henningsson, Dahllöf (2003) [48]	To assess the effects of Palatal Plate Therapy (PPT) on oral motor function, articulation and communication preferences after 4 years of therapy.	Use of a palatal expander with orofacial stimulation.
Finestack, O’Brien, Hyppa-Martin and Lyrek (2017) [49]	To evaluate the quality of an intervention focused on improving the narrative skills of children with DS, using an approach that includes visual supports.	Sessions of between 30 and 60 min three times a week. Game-based learning
Goetz, Hulme, Brigstocke, Carroll, Nasir and Snowling (2008) [50]	To assess whether children with DS benefit from an intervention program that trains phonological awareness, letter knowledge and speech production.	Phonological intervention program of 5 weekly sessions for 16 weeks (8 for precursor literacy skills and 8 for literacy)
Burgoyne, Duff, Snowling, Buckley and Hulme (2013) [51]	To evaluate the efficacy of a 6-week teaching program aimed at developing phoneme blending skills in children with DS.	Individual daily 10–15 min intervention sessions on phonological awareness skills
Regis, Lima, Almeida, Alves and Delgado (2018) [33]	To analyze the contributions of speech therapy to the language development of children with DS	8 therapy sessions
Yoder, Woynaroski, Fey and Warren (2014) [52]	To assess the effectiveness of Milieu Communication Teaching (MCT) based on the frequency of intervention in subjects with DS	Milieu Communication Teaching (prelinguistic milieu teaching, milieu language teaching and responsivity education)
Martín-Urda, Carchenilla and Moraleda (2019) [53]	To determine if there are improvements in morphology, syntax, pragmatics and semantics with a non-systematized intervention in children with DS	Non-systematized speech therapy intervention of two sessions of 40 min a week for 5 years.

**Table 4:** Assessment instruments in the articles consulted.

Authors/Year	Instruments Used
van Bysterveldt, Gillon and Foster-Cohen (2010) [40]	Peabody Picture Vocabulary Test—III, Pre-School Language Scale—Fourth Edition and Hodson’s Assessment of Phonological Patterns, Third Edition
Camarata, Yoder and Camarata (2006) [41]	Spontaneous speech samples
Lemons, King, Davidson, Puranik, Al Otaiba and Fidler (2018) [42]	Ad hoc survey
Burgoyne et al. (2012) [43]	WPPSI-III, Early Word Recognition (EWR) from the York Assessment of Reading for Comprehension (YARC) Early Reading battery
Linn et al. (2019) [44]	Communicative Development Inventory (CDI) adapted to people with DS (CDI-DS) and Bayley III Test
Barbosa, Lima, Alves and Delgado (2018) [45]	Self-made questionnaire and discourse analysis
Naess (2016) [46]	Experimental syllable-initial, syllable-final, rhyme and phoneme-initial matching tasks
Lemons et al. (2015) [47]	Leiter-R Brief IQ and Woodcock-Johnson III Test of Achievement
Sepúlveda, López-Villaseñor and Heinze (2013) [31]	Objective and Criterial Language Battery (BLOC)
Wright, Kaiser, Reikowsky and Roberts (2013) [28]	Analysis of a corpus of videos
Yoder, Woynaroski, Fey, Warren and Gardner (2015) [29]	Ad hoc questionnaire completed by the parents
Carlstedt, Henningsson, Dahllöf (2003) [48]	Ad Hoc assessment of articulation and myofunctional assessment with Ad Hoc protocol
Finestack, O’Brien, Hyppa-Martin and Lyrek (2017) [49]	Differential Ability Scales II (DAS-II), CELF-IV Clinical Evaluation of Language Fundamentals, Test of Narrative Language (TNL) and Conversational Language Sample
Goetz, Hulme, Brigstocke, Carroll, Nasir and Snowling (2008) [50]	British Picture Vocabulary Scales, Nonverbal IQ, British Ability Scales II
Burgoyne, Duff, Snowling, Buckley and Hulme (2013) [51]	YARC Early Word Recognition (EWR) test, Expressive and Receptive One Word Picture Vocabulary Test, Teaching Assistant Questionnaire
Regis, Lima, Almeida, Alves and Delgado (2018) [33]	Ad Hoc evaluation guideline
Yoder, Woynaroski, Fey and Warren (2014) [52]	Mental Development Index, Bayley II, Screening Tool for Autism in 2-year-olds
Martín-Urda, Carchenilla and Moraleda (2019) [53]	WISC-IV Intelligence Scales Battery of Objective and Criterial Language in its computerized version (BLOC-INFO)

## Data Availability

Not applicable.
